# The Proteasome and the Delicate Balance between Destruction and Rescue

**DOI:** 10.1371/journal.pbio.0020013

**Published:** 2004-01-20

**Authors:** Michael H Glickman, Noam Adir

## Abstract

The proteasome is a large multiprotein complex that degrades unwanted cellular proteins. The mechanisms that control this protein-eating machine are being uncovered

Inside eukaryotic cells there is a massive protein complex called the proteasome whose raison d'être is to remove unnecessary proteins by breaking them down into short peptides. The proteasome is thus responsible for an important aspect of cellular regulation because the timely and controlled proteolysis of key cellular factors regulates numerous biological processes such as cell cycle, differentiation, stress response, neuronal morphogenesis, cell surface receptor modulation, secretion, DNA repair, transcriptional regulation, long-term memory, circadian rhythms, immune response, and biogenesis of organelles (Glickman and Ciechanover 2002). With the multitude of substrates targeted and the myriad processes involved, it is not surprising that aberrations in the pathway are implicated in the pathogenesis of many diseases, including cancer.

With so many proteins to target for degradation, the activity of the proteasome is subject to multiple levels of regulation. In the overwhelming majority of cases, selected proteins are first “labeled” by the addition of several copies of a small protein tag called ubiquitin and are thus targeted for degradation in the proteasome (Figure 1). The ubiquitination of proteins is regulated through precise selection of protein substrates by specific E3 ubiquitin ligases (Pickart 2001). These enzyme complexes each recognize a subset of substrates and tag them by linking the carboxyl terminus of ubiquitin with an amino group on the target protein via an amide bond (Figure 1).

**Figure 1:**
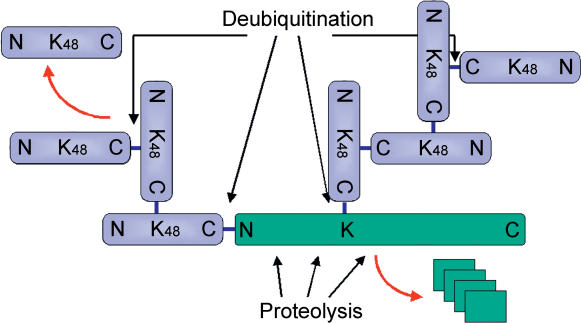
Structure of an Ubiquitinated Protein Ubiquitin (light violet) is a small 76 amino acid protein that can be covalently attached to target proteins (green) by specific E3 ubiquitin ligases. Such conjugation takes the form of an isopeptide bond between the carboxyl terminus of ubiquitin (denoted as C) and a lysine amino sidechain (K) on the substrate, or in some cases, conjugation can be via a peptide bond between ubiquitin and the amino terminus of the protein (N). These amide bonds are indicated as blue links. Multiple ubiquitin moieties can link in a similar manner via lysine-48 (K48) to form a polyubiquitin chain. As symbolized, more than one such chain can assemble on a single target. The result is a branched fusion protein with multiple amino termini (seven in the depicted example) coalescing at a single carboxyl terminus. Polyubiquitination in this manner targets proteins to the proteasome, where they are hydrolyzed into short peptides (green stack). Deubiquitinating enzymes can hydrolyze the bond between one ubiquitin moiety and another or between ubiquitin and the target protein.

Interestingly, ubiquitination is a reversible process. Even when a protein has been tagged with ubiquitin, its fate is not sealed—specific hydrolytic enzymes called deubiquitinases can remove the ubiquitin label intact (Figure 1). By deubiquitinating their substrates, these enzymes compete with the proteasome, which acts on the polyubiquitined form. In the competition between proteolysis and deubiquitination, polyubiquitinated proteins rarely accumulate in the cytoplasm of “healthy” cells, as they are either irreversibly degraded or deubiquitinated and rescued. It is thought that this competition provides a certain level of stringency or quality control to the system. Based on sequence homology, deubiquitinating enzymes were traditionally classified into two families: ubiquitin-specific proteases (UBPs or USPs) and ubiquitin carboxy-terminal hydrolases (UCHs). Both enzyme families are classified as cysteine proteases that employ an active site thiol to cleave ubiquitin from its target (Kim et al. 2003; Wing 2003).

The proteasome itself is made up of a multiprotein core particle (CP) where proteolysis occurs and a separate multiprotein regulatory particle (RP) that recognizes and prepares substrates for degradation by the CP. A base subcomplex of the RP is pivotal in anchoring polyubiquitin chains during this process, either directly or via auxiliary ubiquitin-binding proteins (Lam et al. 2002; Hartmann-Petersen et al. 2003). The base attaches to the outer surface of the CP and uses energy to unravel the substrate, simultaneously with preparing the channel that leads into the proteolytic chamber of the CP (Forster and Hill 2003). The lid subcomplex of the RP attaches to the base and is required for proteolysis of ubiquitin–protein conjugates, but not of unstructured polypeptides (Glickman et al. 1998; Guterman and Glickman 2003). The size and complexity of this protein-eating machine hints at the exquisite controls that must rgulate its function.

An intriguing evolutionary and structural relationship between the proteasome lid and an independent complex, the COP9 signalosome (CSN), may shed light on their respective roles in regulated protein degradation. Both are made up of eight homologous protein subunits that contain similar structural and functional motifs. While a lot is still unknown, the CSN appears to mediate responses to signals (e.g., light, hormones, adhesion, nutrients, DNA damage) in a manner that is intimately linked to the ubiquitin–proteasome system. This is accomplished, for instance, by suppressing ubiquitin E3 ligase activity or interacting with various components of the pathway (Bech-Otschir et al. 2002; Cope and Deshaies 2003; Li and Deng 2003). In particular, one subunit—Csn5—moderates SCF (Skp1–cullin–F box) and other cullin-based E3 ubiquitin ligases by removal of the ubiquitin-like Rub1/Nedd8 molecule from the cullin subunit of the ligase complex. Further analysis of the CSN will no doubt uncover additional mechanisms whereby ubiquitin-mediated protein degradation is controlled.

Surprisingly, the proteasome itself harbors intrinsic deubiquitination activity (Eytan et al. 1993). Moreover, both the lid and the base contribute independently to RP deubiquitination activity. The source of this activity has been attributed to a number of different subunits. These include the associated cysteine proteases Ubp6/USP14 (Borodovsky et al. 2001; Legget et al. 2002), UCH37/p37 (Lam et al. 1997; Hoelzl et al. 2000), and Doa4/Ubp4 (Papa et al. 1999), as well as the intrinsic proteasome subunit Rpn11/POH1 (Verma et al. 2002; Yao and Cohen 2002). The importance of these components to proteasome function is apparent in their partially overlapping properties. In groundbreaking work, an intrinsic “cryptic” deubiquitinating activity that is sensitive to metal chelators has been reported for the proteasome, in addition to “classic” cysteine protease behavior (Verma et al. 2002; Yao and Cohen 2002). This metalloprotease-like activity maps to the putative catalytic MPN+/JAMM motif of the lid subunit Rpn11 and lies at the heart of proteasome mechanism by linking deubiquitination with protein degradation. Notably, Rpn11 shares close homology with Csn5, which is also responsible for proteolytic activities in its respective complex.

By defining a new family of putative metalloproteases that includes a proteasomal subunit, a CSN subunit, and additional proteins from all domains of life, the MPN^+^/JAMM motif garnered great attention. The trademark of the MPN^+^/JAMM motif is a consensus sequence E—HxHx_(7)_Sx_(2)_D that bears some resemblance to the active site of zinc metalloproteases. Members of this family were predicted to be hydrolytic enzymes, some of which are specific for removal of ubiquitin or ubiquitin-like domains from their targets (Maytal-Kivity et al. 2002; Verma et al. 2002; Yao and Cohen 2002).

In a further development, two independent groups determined the molecular structure of an MPN^+^/JAMM protein from an archaebacterium (Ambroggio et al. 2003; Tran et al. 2003). The structures identify a zinc ion chelated to the two histidines and the aspartic residue of the MPN^+^/JAMM sequence. The fourth ligand appears to be a water molecule activated through interactions with the conserved glutamate to serve as the active site nucleophile. Overall, this protein certainly has properties consistent with a metallohydrolase and can serve as the prototype for the deubiquitinating enzymes in its class. This revelation adds an all-new enzymatic activity and, with it, an additional layer of regulation to the ubiquitin–proteasome system.

Now that it is evident that the proteasome contains a member of a novel metalloprotease family, a fundamental question can be raised: why does a proteolytic enzyme like the proteasome need auxiliary proteases for hydrolysis of ubiquitin domains? At first glance, the delegation of tasks between the proteolytic subunits of the proteasome (situated in the proteolytic core particle) and the auxiliary deubiquitinating enzymes (situated in the regulatory particle) is clear-cut: the latter cleave between ubiquitin domains, while the core proteolytic subunits process the target protein itself (Figure 1). However, this still does not explain the mechanistic rational for finding deubiquitination within the proteasome itself. In principle, deubiquitination could be used for (1) recycling of ubiquitin, (2) abetting degradation by removal of the tightly folded highly stable globular ubiquitin domain, or (3) mitigating degradation by removal of the ubiquitin anchor, without which the substrate is easily released and rescued. There is evidence that recycling of ubiquitin by the proteasome is indeed a crucial feature of deubiquitination in proper cellular maintenance (Legget et al. 2002). Distinguishing between options 2 and 3, however, depends to a large extent on the delicate balance between the two proteolytic activities associated with the proteasome: proteolysis and deubiquitination (Figure 2).

**Figure 2:**
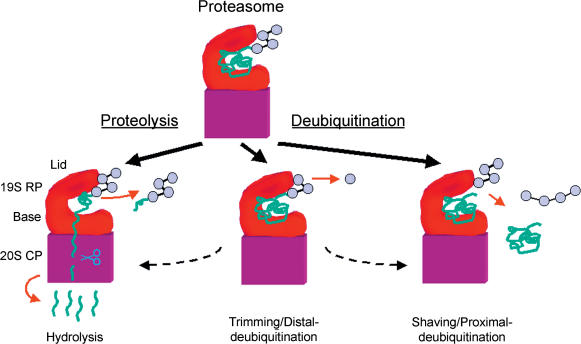
Deubiquitination versus Proteolysis at the Proteasome Once recognized and anchored to the proteasome via its polyubiquitin tag (light violet), a substrate (green) can be unraveled, unfolded, and translocated by the 19S regulatory particle (red) into the proteolytic chamber of the 20S core particle (purple), where it is hydrolyzed into short peptides (left). A byproduct of proteolysis is the polyubiquitin anchor (that may still be linked to a residual peptide). Cytoplasmic deubiquitinating enzymes eventually process this chain and recycle ubiquitin. However, the proteasome can also directly deubiquitinate the substrate, with diverse outcomes. For example, the substrate can be “shaved” upon cleavage of the bond to the proximal ubiquitin (right). Without its anchor, the substrate is presumably released and rescued. A distinct deubiquitinating activity is “trimming” or removal of the distal ubiquitin moiety (middle). According to one hypothesis, trimming serves as a timer; extended or difficult-to-process chains allow ample time for substrate unfolding and irreversible proteolysis (left), while short or easy-to-process chains inevitably lead to substrate release and rescue (right). This delicate balance between destruction and rescue is fundamental to proteasome efficiency.

Once bound to the proteasome, a polyubiquitinated substrate can be unfolded by the RP and irreversibly translocated into the CP. It has been proposed that long polyubiquitin chains commit a substrate to unfolding and degradation by the proteasome, whereas short chains are poor substrates because they are edited by deubiquitinating enzymes, resulting in premature substrate release (Eytan et al. 1993; Lam et al. 1997; Thrower et al. 2000; Guterman and Glickman 2003). Extended polyubiquitin chains could slow down chain disassembly, thereby allowing ample time for unfolding and proteolysis of the substrate (Figure 2). Interestingly, both “trimming” and “shaving” deubiquitinating activities are associated with the proteasome, though the exact contribution of the various proteasome-associated deubiquitinating enzymes to each of these distinct activities has yet to be elucidated. It is expected that in order to obtain efficient proteolysis of the target, shaving of chains at their proximal ubiquitin should be slower than the rate of trimming at the distal moiety. As an outcome of this requirement, longer polyubiquitin tags would be preferential substrates for degradation by the proteasome. Thus, the uniqueness of ubiquitin as a label for degradation may lie in its being a reversible tag. Deubiquitinases, such as Rpn11, serve as proofreading devices for reversal of fortune at various stages of the process, right up to the final step before irreversible degradation by the proteasome.

Identifying Rpn11 and Csn5 as members of a novel class of metallohydrolases immediately elevates them into promising “drugable” candidates. Undoubtedly, the molecular structures deciphered by the groups of Deshaies (Ambroggio et al. 2003) and Bycroft (Tran et al. 2003) will focus efforts to design novel site-specific inhibitors of the ubiquitin–proteasome pathway. While Csn5 is thought to impede the action of ubiquitin ligases through shaving cullins from their Rub1/Nedd8 modification (and possibly also by deubiquitinating substrates bound to the cullins), the outcome of Rpn11 inhibition will depend largely on whether Rpn11 participates primarily in shaving substrates from their chains, promoting release and rescue, or in trimming the polyubiquitin tag, allowing for proteolysis quality control (Figure 2).

## Abbreviations

CP: core particle
CSN: COP9 signalo-some
RP: regulatory particle
SCF: Skp1–cullin–F box
UBP: ubiquitin-binding protein
UCH: ubiquitin carboxy-terminal hydrolase
UBP/USP: ubiquitin-specific protease
